# “I feel like I'm in a dream”: a qualitative investigation of the lived experience of Visual Snow Syndrome

**DOI:** 10.3389/fneur.2026.1973126

**Published:** 2026-10-08

**Authors:** Amy Claire Thompson, Georgia F. Caruana, Jacob M. Paul, Olivia Carter, Jason D. Forte

**Affiliations:** 1 Melbourne School of Psychological Sciences, University of Melbourne, Parkville, VIC, Australia; 2 Department of Psychiatry, University of Melbourne, Parkville, VIC, Australia

**Keywords:** chronic illness, lived experience, quality of life, visual snow, Visual Snow Syndrome

## Abstract

**Background:**

Visual Snow Syndrome (VSS) is neurological condition typified by persistent visual snow, or static, throughout the visual field, alongside additional phenomena that impact night vision, visual perception, and visual processing. Only formally characterized in the 2010s, little consideration has been given to the broader impacts of this condition on the lives of those who experience it. This study presents the first qualitative investigation of the lived experience of Visual Snow Syndrome, to further inform clinical, research, and consumer communities alike.

**Methods:**

Forty-nine adults who self-identified as having VSS completed an online survey that queried the good and bad aspects of VSS, and its general impact on their daily life. Their qualitative data were analyzed iteratively, to determine the key themes represented across and between individual responses.

**Results:**

The lived experience of VSS varied both inter and intra-individually across this sample. Responses endorsed several themes: mental health and psychosocial wellbeing, functional vision, the dynamic course of visual snow syndrome, clinical challenges, and medical system limitations. The majority of participants described their VSS as a negative component of their daily life, with specific descriptions outlining how living with VSS worsened their mental health, cognition, sleep, daily routines, visual quality, and comfort in both natural and artificial light. However, one third of participants were able to identify some positive aspects of their visual snow experience, highlighting that it can bring unique meaning and fascination to their lives.

**Conclusion:**

VSS has broad and varied impacts on the day-to-day lives of those who experience it. It can introduce substantial mental health and psychosocial wellbeing challenges and functional vision impairments, but also a unique, and sometimes beautiful perspective. These experiences, paired with its variability between and within persons, indicates a need for more holistic and individualized approaches to the long-term treatments and supports.

## Introduction

Visual Snow Syndrome (VSS) is a neurological condition characterized by persistent visual snow, or static, throughout the visual field, along with additional symptoms including palinopsia, enhanced entoptic phenomena, and nyctalopia (poor night vision)

(1). The clinical presentation of VSS is heterogeneous: the visual phenomena vary considerably between people, in their presence, frequency, and sometimes expression. Migraine, tinnitus, and a range of mental health conditions are commonly reported comorbidities (2, 3). The underlying pathophysiology of VSS remains uncertain, although converging evidence implicates altered processing across visual and wider cortical networks (4). Since VSS was first formally characterized in 2014 (5), considerable research has therefore been directed toward defining its clinical phenotype and understanding its neurobiological basis. In contrast, there has been little consideration given to the impact of VSS on the lives of people who live with it.

To date, no qualitative research has investigated the lived experience of VSS. Much of our understanding of life with VSS is derived from free-text questions included in some questionnaire-based research; from the limited qualitative data available from chart review studies; or from patients' unprompted descriptions. Many of these data have related to people who have sought medical treatment for their VSS, or who otherwise find their VSS to be distressing to varying degrees. Understandably, this has led to an assumption in the literature that VSS is an experience characterized by suffering and disability. Indeed, at least five separate research papers refer to people with VSS as “VSS sufferers” or state that “patients suffer from VSS”, although it is not clear whether this terminology is truly representative of the experience of everyone with VSS (6–10).

There is also growing evidence that a proportion of people with VSS are completely unaware that they live with the condition (11–15). In recent years, a number of works have assessed experiences of VSS in the general population, and uncovered a subset of people who have seemingly never sought help for, or otherwise investigated, their visual phenomena. There is also quantitative evidence to show that people who have had VSS their whole lives experience better health-related quality of life, and find the condition to be less impactful overall, despite experiencing the same number of symptoms with the same frequency as people who develop the condition later in life (3, 12). For example, in their 2021 study on the psychiatric symptoms associated with VSS, Solly et al. probed health-related quality of life in people with VSS. Everyone who participated in this study was able to identify at least one area of their life which was impacted by VSS, although some participants (mostly with lifelong VSS) provided the caveat that the impacts were minimal (3). Additionally, Costa et al. (15) found that visual snow (the symptom, not the syndrome) was related to attentional absorption, and that many people in a large non-clinical sample found their visual snow to be fascinating, rather than distressing.

In this study, we present the first qualitative investigation of the lived experience of Visual Snow Syndrome. We aim to present a holistic overview of the experience of VSS, including understanding aspects of the condition which people find to be upsetting or not, and how it impacts the lives of people who live with it. Because the purpose of this study was to characterize the breadth of lived experience rather than identify clinical predictors of experience, our analyses focused on themes expressed across participants rather than subgroup comparisons. Questions about how lived experience differs according to symptom profile or comorbidities represent important directions for future, hypothesis-driven quantitative and mixed-methods research.

This study presents a rich dataset of varied VSS experiences. We hope this will enhance researchers' and clinicians' understanding of the lived experience of VSS, helping to guide future research, experimental design, and clinical practice in both the assessment and treatment of those who experience VSS—whatever their experience of the condition may be.

## Methods

This study was approved by the University of Melbourne Human Research Ethics Committee and was conducted in accordance with the Declarations of Helsinki. Participants provided informed consent via an online form prior to commencement of the study. No funding was received toward this study.

These data form part of a larger study, and were derived from the baseline intake questionnaire. Only measures relevant to this study are reported here.

### Participants

Between May and October 2024, we recruited 49 participants who self-identified as experiencing VSS from online communities related to VSS and from mailing lists of previous study participants. We targeted online communities on Facebook and Reddit, including those specific to people with lifelong VSS experiences.

#### Response screening

Participant responses were screened to ensure that responses were made in good faith, as described in Thompson et al. (14). No participants were excluded.

### Measures

Participants completed a series of questionnaires in Qualtrics (16). First, they were screened for the presence, frequency, and perceived impact of perceptual phenomena associated with VSS using the long-form Melbourne Visual Snow Questionnaire (MVSQ) (14). This is a previously-reported measure designed for researchers to assess VSS symptoms and determine whether research participants likely meet the ICHD criteria for VSS. Symptoms are deliberately described in language which is accessible without prior knowledge of VSS, and the questionnaire probes both presence and frequency of symptoms, as well as onset timing (if known), and possible alternative explanations for symptoms (such as migraine with aura or HPPD). Participants also completed the Visual Snow Handicap Inventory (VSHI; a measure of the distress which results from visual snow) (17), the Depression, Anxiety and Stress Scale (21-item version; DASS-21) (18), and various other measures which are not relevant to the present study.

We also included several optional qualitative questions to assess participants' lived experience of VSS. These questions included: (1) “*Please describe how the static in your vision impacts your life*”; (2) “*Is your Visual Snow Syndrome sometimes a bad thing? What is bad about your Visual Snow Syndrome?*”; (3) “*Is your Visual Snow Syndrome sometimes a good thing? What is good about your Visual Snow Syndrome?*”; and other questions not relevant to this paper.

### Participant categorization

As participants' prior medical diagnoses could not be independently verified, they were placed into prospective diagnostic categories according to the ICHD diagnostic criteria for VSS based on their responses to the MVSQ (1). The specific categorization process is outlined in Thompson et al. (12). Categories included lifelong VSS (VSS which began in early childhood or which has been experienced for as long as the participant could remember); acquired VSS (VSS which developed later in life, or when the participant can remember a time before they experienced VSS); possible Hallucinogen Persisting Perception Disorder (HPPD; where drug use immediately preceded the onset of symptoms); visual snow in the absence of sufficient additional symptoms to merit VSS diagnosis; and additional symptoms in the absence of persistent visual snow.

#### Missingness

There were no missing data. Some participants elected to respond “N/A” or “no answer” to some questions.

### Software and descriptive statistics

Scale scoring and descriptive statistics were calculated using R 4.4.3 (19).

### Qualitative content analysis

In keeping with our aim to present an overview of the lived experience of VSS, we focused on the qualitative questions described above, which queried the good, bad and overarching impacts of living with VSS. Responses to these questions were analyzed by authors ACT and GFC using inductive qualitative content analysis, following the systematic approach described by Erlingsson and Brysiewicz (20).

For each question, anonymized participant responses were randomly divided between the two authors for initial analysis. Each author independently reviewed their allocated responses and identified relevant *meaning units*, which were condensed while preserving their core *condensed meaning units*. These were subsequently reviewed and discussed jointly by ACT and GFC to ensure consistency in interpretation and terminology.

The authors then collaboratively assigned *codes* that succinctly described the manifest content of each condensed meaning unit, beginning to establish common connections between the individual responses. Each code was then discussed, compared, and appraised, so that those reflecting the same content or context could be grouped into “*categories”*. Finally, “*themes”* were established, by grouping categories that reflected concordant latent content. Analysis proceeded iteratively, with codes, categories and themes repeatedly reviewed against the underlying participant responses and refined through discussion between the two authors. Figure 1 provides an overview of this process.

**Figure 1:**
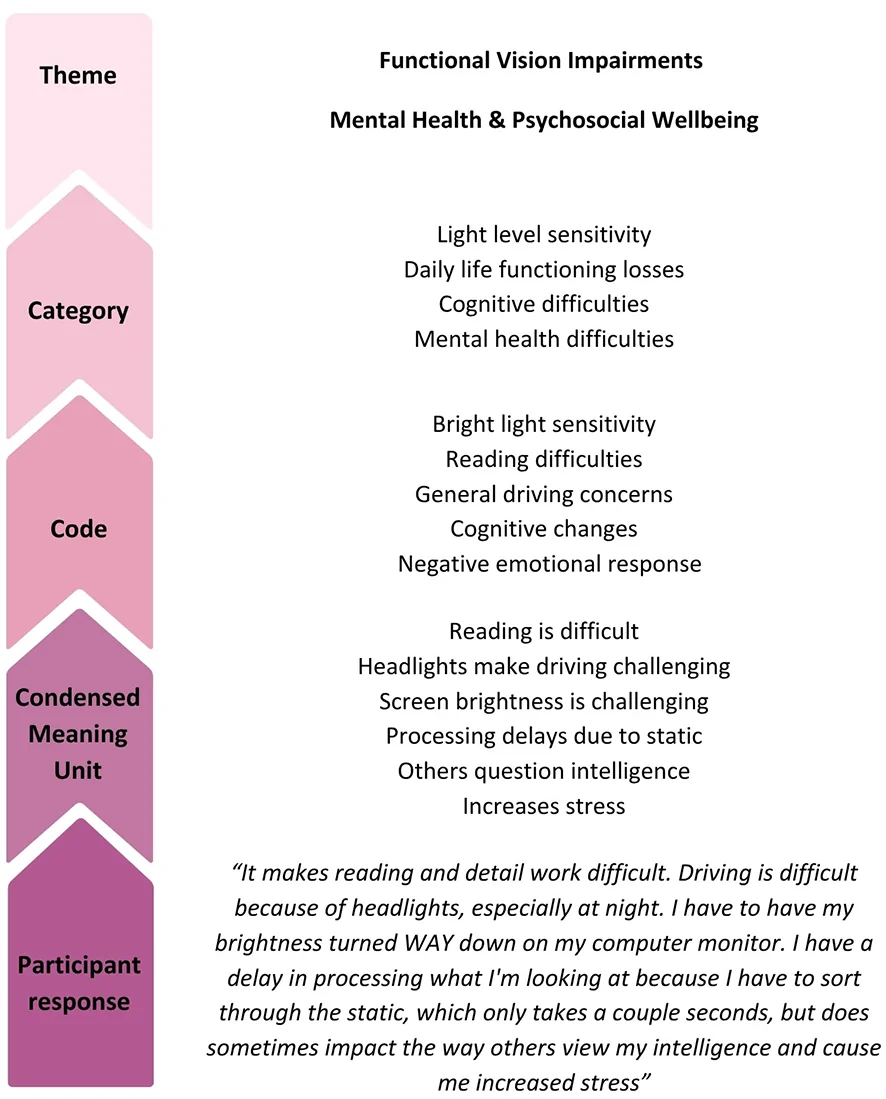
Example of the qualitative content analysis pipeline. Each participant response was reviewed in a stepwise manner to determine the key theme/s it would ultimately reflect. Responses often described multiple concepts; thus, a single response could endorse multiple themes.

## Results

### Participant characteristics

Demographic, visual phenomena, and mental health characteristics of the sample are presented in Table 1. Participants' mean age was 32 years (*SD* = 11), and most identified as female. Of the 49 participants, 18 had lifelong VSS and 20 acquired, five had possible HPPD, and five had visual snow in the absence of sufficient additional symptoms for VSS diagnosis. All participants were included in our analyses regardless of their diagnostic categorization and whether or not they seemingly met the ICHD criteria for VSS. Participants' mean VSHI score was 47.92 (*SD* = 25.05), indicating that this sample experiences moderate “handicap” as a consequence of visual snow. Despite over half of our participants disclosing a diagnosis of anxiety and 49% disclosing a diagnosis of depression at some point in their lives, current DASS-21 scores tended to be in the normal range.

**Table 1:** Summary of sample characteristics.

	*N* = 49
Demographic data	
Age (mean, SD)	32.04, 11.01
Gender (female, male, non-binary)	30, 17, 2
Probable migraine (no aura)	12
Probable migraine (aura)	24
Drug history	27
Tinnitus	44
Visual snow experiences	
VSS (lifelong, acquired)	18, 20
Possible HPPD	5
Visual Snow in the absence of sufficient additional symptoms for VSS diagnosis	5
Additional symptoms in the absence of persistent Visual Snow	1
Medical diagnosis of VSS	24
Visual phenomena	
Palinopsia	30
Excessive floaters	44
Blue field entoptic phenomenon (BFEP)	34
Self-light of the eyes	35
Other entoptic phenomena	29
Self-report photophobia	40
Nyctalopia	43
Mental health	
Anxiety (diagnosed, experienced but not diagnosed)	28, 15
Depression (diagnosed, experienced but not diagnosed)	24, 17
DASS-21 depression score (mean, SD)	5.24, 3.53
DASS-21 anxiety score (mean, SD)	5.92, 4.96
DASS-21 stress score (mean, SD)	7.86, 3.88
Visual snow severity measures (mean, SD)	
Total additional symptoms (i.e., not visual snow itself)	6.08, 1.64
VSHI score	47.92, 25.05

Unless otherwise specified, data are counts. The one participant who experiences additional symptoms in the absence of visual snow has received a medical diagnosis of VSS. DASS-21 mean scores are all within normal ranges, i.e., do not indicate clinical depression, anxiety or stress. The mean VSHI score indicates moderate handicap as a consequence of VSS.

### Qualitative outcomes

Responses reflected several themes and subcategories, with *mental health and psychosocial wellbein*g and *functional vision* outcomes emerging as common concepts across all three questions. Table 2 summarizes each theme, category and code, and the percentage of respondents who endorsed this theme. Figure 2 provides an additional overview of the major experiences endorsed by participants across all questions.

**Figure 2:**
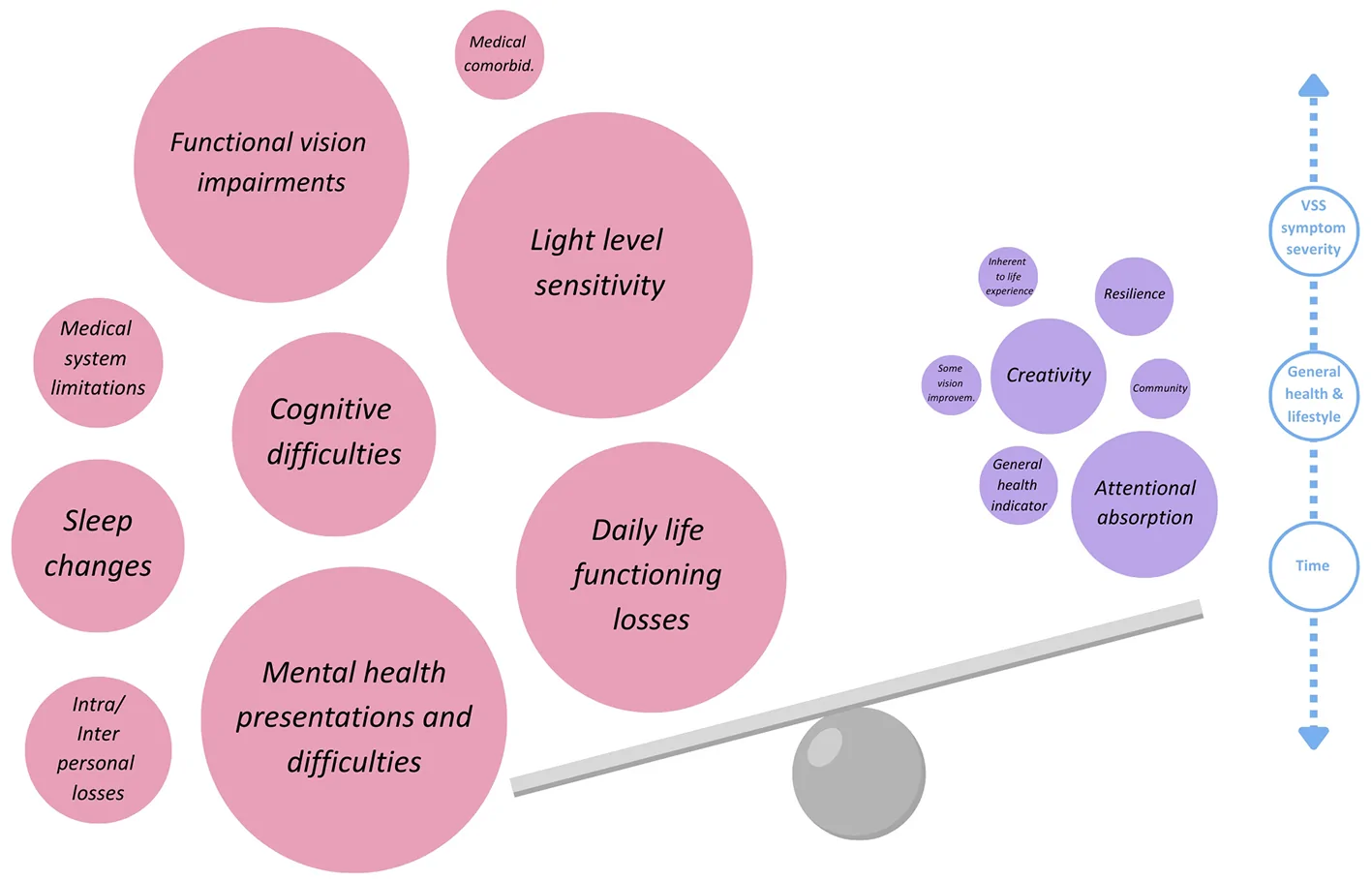
Overview of the major outcomes participants reported as a result of their experience living with VSS. Negative experiences are depicted in pink, and positive in purple, with the size of the experience representing the prevalence of it within the sample. Whilst participants did report some positive outcomes of living with VSS, the impacts are imbalanced to be predominantly more negative. As per the blue bi-directional arrow, participants indicated that their experience of these outcomes can be dynamic, varying based on their VSS symptom severity, general health and lifestyle, and simply over time.

**Table 2:** Summary of all themes, categories, and codes representing the impacts, bad things, and good things about living with Visual Snow Syndrome.

2.A. If you are willing, please describe how the static in your vision impacts your life	2.B. Is your Visual Snow Syndrome sometimes a bad thing? What is bad about your Visual Snow Syndrome?	2.C. Is your Visual Snow Syndrome sometimes a good thing? What is good about your Visual Snow Syndrome?
Theme 1—mental health and psychosocial wellbeing (*n =* 29, 65.91%) *Mental Health−15* responses describing ° Anxiety ° Constant negative feelings ° Detachment ° Reduced quality of life ° Sadness *Cognition—three* responses describing ° Distractibility *Daily life functioning−12* responses describing ° Device use challenges ° Driving difficulty ° Hobby loss ° Reading impacts *Sleep and fatigue—six* responses describing ° Sleep changes ° Constant tiredness *Positive wellbeing−1* response describing ° Positive emotional response	Theme 1—mental health and psychosocial wellbeing (*n* = 40, 81.63%) *Mental health difficulties−23* responses describing ° Fear inducing ° Health anxiety ° Hopelessness ° Negative emotional response ° Exhaustion/fatigue ° Depersonalization ° Derealization ° Shame *Cognitive difficulties−15* responses describing ° Irritability ° Sensory overload ° Attentional difficulties ° Can't relax ° Brain fog ° General cognitive changes ° Distracting *Daily-life functioning losses−9* responses describing ° Reading difficulties ° Night driving difficulties ° General driving concerns ° Loss of hobby enjoyment ° Limited career development *Inter and intrapersonal social losses−7* responses describing ° Loss of independence ° Makes life harder ° Inconvenience ° Being misunderstood ° Social disadvantages *Sleep changes – 4* responses describing ° Negative impact on sleep	Theme 1—nothing good or positive (*n* = 26, 54.17%) *Nothing good or positive−26* responses describing ° Just nothing good
Theme 2—functional vision impacts (*n =* 28, 64.64%) *Vision impacts−13* responses describing ° Reduced visual quality and clarity ° Depth perception changes ° Lack of visual contrast *Lighting impacts−18* responses describing ° Artificial light difficulty ° Bright light difficulty ° Low light difficulty ° Difficulty in any light extremes ° Environmental interference	Theme 2—functional vision impairments (*n =* 33, 67.35%) *Vision losses−11* responses describing ° Loss of previous visual experience ° Lack of clear vision ° Depth perception difficulties *Light level sensitivity−18* responses describing ° Sensitive to sunlight ° Bright light sensitivity ° Inability to look at/enjoy nature ° Light level challenges ° Poor night vision ° Light related pain	Theme 2—mental health and psychosocial wellbeing (*n =* 13, 27.08%) *Attentional absorption—eight* responses describing ° Provides distraction ° Generates fascination ° Can be beautiful ° Unique perspective of the world ° Vivid dreams ° Positive visual hallucinations *Creativity—four* responses describing ° Increased creativity *Resilience—three* responses describing ° Motivating ° Increases resilience ° Positive retrospection *Community—one* response describing ° Created new social connections
Theme 3—dynamic course and impacts (*n =* 10, 22.73%) *Neutral response—seven* responses mentioning ° It's normal ° Mild annoyance ° Symptoms fluctuate *Impact decreases—three* responses mentioning ° Gets easier	Theme 3—clinical challenges (*n =* 5, 10.20%) *Prognostic concerns—five* responses describing ° Future related anxiety ° Concerns about symptom progression ° Concerns about blindness *Medical comorbidities—one* response describing ° Migraine comorbidity ° Tinnitus comorbidity	Theme 3—not necessarily good; more neutral (*n* = 5, 10.42%) *Neutral response—two* responses describing ° Current level manageable ° Bearable *Inherent to my life experience—two* responses describing ° Would want to keep it ° Part of me *Facilitates concessions—two* responses describing ° Can acquire disability concession ° Avoidance mechanism *Health indicator—three* responses describing ° Provides general health indications
	Theme 4—medical system limitations (*n* = 4, 8.16%) *Negative experiences with medical system—four* responses describing ° Lack of medical understanding ° Diagnostic difficulties ° Discomfort with medical professionals ° Lack of treatment options	Theme 4—functional improvements (*n* = 2, 4.17%) *Vision improvements—two* responses describing ° Increased visual acuity ° Increased attention to detail
	Theme 5—Not necessarily bad; more neutral (*n* = 4, 8.16%) *Inherent to my life experience—four =* responses describing ° Central to personal identity ° Sometimes ignorable ° General inconvenience

Table outlines the theme and comprising *categories* that derived from each question posed to participants, with dot point examples of the general content that is encompassed by each category. A single participant response often endorsed, and was thus counted across multiple themes, as reflected by *n* and percentage statistics exceeding the total sample size of *N* = 49. Within the endorsement of each theme, participant responses often described content relevant to multiple categories. Five participants elected not to respond to Question 1, one participant elected to not respond to Question 3.

Note that participant responses have not been edited. They are presented as they were provided and as such, linguistic and grammar choices are those of our participants. Participants all confirmed that they were fluent in English, but they were not all native speakers.

#### Question one—If you are willing, please describe how the static in your vision impacts your life

Three themes arose from this question (see Tables 2.A, 3). Negative impacts of VSS on *mental health and psychosocial wellbeing* were endorsed by 65.91% of participants. These responses described recurrent and persistent feelings of fear, anxiety, and sadness, and an overarching reduction in perceived quality of life. Example responses included, “*It makes me rather anxious. Not all the time, the associated anxiety rises and falls in severity, sometimes I will get very anxious about it and other times it will not bother me, seemingly randomly*”, and “*It impacts my visual quality (I'll never see clearly), which plays on my mind and degrades my quality of life. This subsequently leads to a significant negative impact on my mental health, and has on more than one occasion lead to a severe decline in mental wellbeing*”.

**Table 3:** Representative quotes describing the *impact* of Visual Snow Syndrome on daily life.

Theme 1. Mental health and psychosocial wellbeing	
Mental health	° “It makes me really scared and anxious, feels like I'm in a dream or I'm not real” ° “The most annoying thing, no escape from it especially in the evening when there's not as much light” ° “I get depersonalized when I have a lot of static in my vision. I get really nervous and I get a lot of anxiety” ° “It makes me rather anxious. Not all the time, the associated anxiety rises and falls in severity, sometimes I will get very anxious about it and other times it will not bother me, seemingly randomly” ° “I got used to it, but at the beginning I couldn't sleep anymore or go outside, I had panic attacks” ° “It impacts my visual quality (I'll never see clearly), which plays on my mind and degrades my quality of life. This subsequently leads to a significant negative impact on my mental health, and has on more than one occasion lead to a severe decline in mental wellbeing”
Cognition	° “It can make it difficult to focus and sometimes difficult to see. I find it easier to see when walls are painted dark than white” ° “Distracting, distressing, difficulty focusing on things in front of me (screens, text), difficulty concentrating”
Daily life functioning	° “It makes reading, especially on screens difficult and effortful. Photography is one of my primary hobbies and it affects my ability to determine when objects in the viewfinder are in focus or not. It affects my ability to enjoy nighttime recreation” ° “It is often unsafe to drive because the static is distracting, painfully bright or causes me be practically blind if it”
Sleep and fatigue	° “I think it makes me more fatigued, especially when looking at screens or driving”
Positive wellbeing	° “I feel like “impacts” is kind of vague/broad and maybe that's intentional. I don't really think it negatively effects my life but is nonetheless an absolute constant in it. I feel that I consider it and other visual phenomenon to be a strange quirk to my identity”
**Theme 2. Functional vision impacts**	
Vision impacts	° “It makes it difficult to read and see words. I can't see stars at night. I find it really difficult to be in a room with patterns on the walls, carpets or curtains. I find it difficult when it is really bright. It can be hard to tell if it is raining” ° “I can't see well at night at all. It also ruins my depth perception to a degree, especially when I look at the sky” ° “Usually when it comes to reading things or looking at scenery. It adds a lot of clutter and can make it hard to focus on what I'm trying to look at” ° “I feel like my view of the world is at a lower resolution than everyone else's”
Lighting impacts	° “Sometimes daylight is “wrong”, I see all of this and the floaters and that makes depressive” ° “Symptoms only problematic in last few months which change of job/lighting conditions in new place of work (e.g., no natural light, dim/low artificial lighting and neutral walls/tones in office)” ° “Usually in the nights, it is harder to see and I need to concentrate more. During the day it takes a lot of focus and I get tired more easily”
**Theme 3. Dynamic course and impacts**	
Neutral response	° “I was born with it so it's just normal and doesn't stop me from doing anything.” ° “It depends because I've had this since for 7 years and only in the past year have the symptoms worsened randomly. So when I notice it getting worse then I stress out otherwise I just move on”
Impact decreases	° “It used to make me worry, but it has not for many years” ° “I was diagnosed over two years ago so I've gotten used to it - it definitely affected me when it started”

Participants reported experiencing cognitive, sleep-related and daily life functioning impacts, such as distractibility and fatigue, and a need to alter daily routines due to impacts on hobbies and responsibilities (e.g., driving, reading, digital device use). Such responses described VSS to be “*Distracting, distressing, difficulty focusing on things in front of me (screens, text), difficulty concentrating*”, and reported that it “*makes reading, especially on screens difficult and effortful. Photography is one of my primary hobbies and it affects my ability to determine when objects in the viewfinder are in focus or not. It affects my ability to enjoy nighttime recreation*”.

*Functional vision impacts* emerged as another key theme, experienced by 63.64% of participants. Impacts on functional vision involved changes to visual experiences, including a perceived reduction in visual quality and clarity, depth perception changes, and a lack of visual contrast. The distinct impact of lighting, particularly extreme lighting (very bright and/or very low) were repeatedly described, in the context of both artificial and natural light: “*It makes it difficult to read and see words. I can't see stars at night. I find it really difficult to be in a room with patterns on the walls, carpets or curtains. I find it difficult when it is really bright. It can be hard to tell if it is raining*”; “*Usually in the nights, it is harder to see and I need to concentrate more. During the day it takes a lot of focus and I get tired more easily*.”

Finally, 22.73% of responses described impacts related to the *dynamic course* of VSS. This theme reflected a more neutral and normalized experience of VSS, that remains a mild annoyance, or only becomes impactful upon symptom fluctuation. It featured accounts such as, “*it depends because I've had this since for 7 years and only in the past year have the symptoms worsened randomly. So when I notice it getting worse then I stress out otherwise I just move on”*.

Five participants chose not to disclose any ways in which the VSS impacted their lives.

#### Question two—Is your Visual Snow Syndrome sometimes a bad thing? What is bad about your Visual Snow Syndrome?

Five themes emerged from this question (see Tables 2.B, 4). Again, the most prevalent was *mental health and psychosocial wellbeing*, endorsed by 81.63% of participants. Concordant with question one, the bad experiences accounted for here included fear, anxiety, hopelessness, sadness, and shame. Responses described that “*it always seems bad because it makes me feel unreal and spikes my anxiety and stress a lot, then when I get more stressed it all gets even worse like a cycle. It distracts me and scares me*”; and “*it feels like I'm not living my life. Everything feels fake and I can't live my life like it was before. I feel like a blind person. It's giving me bad depression and anxiety*”.

**Table 4:** Representative quotes describing what is *bad* about Visual Snow Syndrome.

Theme 1. Mental health and psychosocial wellbeing	
Mental health difficulties	° “It feels like I'm not living my life. Everything feels fake and I can't live my life like it was before. I feel like a blind person. It's giving me bad depression and anxiety” ° “It always seems bad because it makes me feel unreal and spikes my anxiety and stress a lot, then when I get more stressed it all gets even worse like a cycle. It distracts me and scares me” ° “Always, all of the time, it is the worst. It really is. It made me depressed for a long time”
Daily life functioning losses	° “I don't have as many career opportunities open to me because I cannot reliably drive so I have to work from home. If I wasn't able to find a remote job I would not be able to work. I often find that in addition, I cannot read a book after “using my vision” too much, such as after an 8-hour day working at a computer and I love reading” ° “I love reading and learning but it is so taxing that I often avoid it” ° “My visual snow syndrome can make daily life somewhat difficult, but I have found ways to cope like painting my walls dark at home, using dark mode with gray text and using tinted glasses. Still, I find it difficult to read because I see lines as wavy and text is doubled. It is difficult to focus and see what I am looking at. It is especially difficult to drive at night, as all I can see is grain and after images” ° “It gets in the way, and it makes it difficult to drive. I don't drive at night anymore because I can't see, and the flashing in the sky on sunny days is really distracting” ° “It prevents me from being able to live a fully undeterred life”
Inter and intrapersonal social losses	° “It's just like... the whole package is like a handicap, which makes life harder, sometimes way harder. Cannot function normally like others every day” ° “It is always a bad thing. It is hard to live with and I don't think people realize how hard it is to live with. I often feel very alone as no one else seems to know of it with the exception of the online community” ° “Yes, it impairs my vision so much that I barely go out on my own because there are few perfect weather days where I feel safe driving and I cannot see at all at night. I feel isolated and my independence has been taken from me” ° “I also cannot go do certain activities like go to a concert, the movies, certain plays or orchestras, etc. because of the lighting causing my symptoms to worsen”
Cognitive difficulties	° “At the moment, it's very prominent at work causing distraction throughout the day” ° “Feeling half-distracted a lot, or feeling brain fogged. I wonder if I would've been more successful as a person or in my career without it” ° “Irritability and anxiety and exhaustion play into it. I'm always overstimulated”
Sleep changes	° “I don't see well/often bump into things during the night if I need to get up and don't put a light on. If I do put a light on, residual/after images are then distracting when trying to get back to sleep” ° “There are more serious things, like causing problems falling asleep on nights where I experience more visual stimuli when closing my eyes”
Theme 2. Functional vision impairments	
Vision losses	° “Yes I hate having it I've not always had it I used to have clear vision so I know how my vision is supposed to be” ° “Yes. It decreases my ability to see and perceive the world in a complete way like I used to be able to. It used to cause me extreme distress but I'm doing well right now” ° “It's awful. It's like viewing the world at a lower resolution” ° “The sky is bad to look at (stargazing is kinda confusing sometimes), and I have really bad night vision nor depth perception”
Light level sensitivity	° “Most things about it aren't great. Light issues are not good” ° “I always have to take my sunglasses with me. I can't read texts with high contrasts” ° “Inability to see clearly, experiencing pain on sunny days” ° “Not seeing clearly, not able to look at the sky for longer periods of time and enjoy sunsets, etc.”
Theme 3. Clinical challenges	
Prognostic concerns	° “It's a constant distraction and it makes me worry as it is progressing, day by day it gets a tiny bit worse, it makes me wonder whether I'll go completely blind one day or the after images will last for so long that I won't be able to function at all” ° “The bad thing is the slow progressiveness and noticing new symptoms or areas where it wasn't present. I miss enjoy something simple like looking out at the sea without immediately seeing my white blood cells and this weird erratic pulsing across the whole” ° “Stress, anxiety, constant worry about future”
Medical comorbidities	° “It has had a profound and defining impact upon my existence, and I believe I would have been quite a different person without it, as well as other health issues such as tinnitus and migraines”
Theme 4. Medical system limitations	
Negative experiences with medical system	° “I don't even feel comfortable asking a physician or optometrist because I don't think anyone will know about it and there isn't treatment” ° “Especially, since it took me more than a year to get a proper diagnosis after seeing four neurologists and neuro-ophthalmologists (I did two brain MRI in case I had multiple sclerosis, EEG, scanners and so on) and it is still not recognize so much in my country, so I have to take extra steps with the administration to explain my issue at work” ° “Wondering if I can say I have a “disability” or not and living in the gray zone of a medical mystery and an invisible condition is quite difficult. Explaining it to people, or reminding loved ones of it, while I generally don't mind, can be exhausting after a while”
Theme 5. Not necessarily bad; more neutral	
Inherent to my life experience	° “It can vary from being easily ignored/not noticed to almost completely debilitating, and anything in between” ° “It feels like if it's not a neutral thing, it's a bad thing. It creates a laundry list of inconveniences that make for a frustrating experience” ° “I'm quite neutral toward my VSS, but yeah, it's not fun having with and I miss the life and vision I had before it”

Numerous cognitive difficulties were reported, including sensory overload, brain fog, disrupted sleep, the loss of previously enjoyable daily activities, and challenges in working life. Inter and intrapersonal social losses were expressed, specifically loss of independence, feelings of being misunderstood, and general social disadvantage. For example, one participant stated that “*I also cannot go do certain activities like go to a concert, the movies, certain plays or orchestras, etc. because of the lighting causing my symptoms to worsen*”.

The second theme, *functional vision impacts* was also highly represented, experienced by 67.35%. In line with the impacts noted in question one, participants expressed how a loss of their previous visual experience, a lack of clear vision, and significant light sensitivity were bad aspects of their VSS: “*it's awful. It's like viewing the world at a lower resolution*”, “*inability to see clearly, experiencing pain on sunny days*”, “*I always have to take my sunglasses with me. I can't read texts with high contrasts*”. A smaller proportion of participants endorsed the theme of *clinical challenges* (10.20%), highlighting prognostic concerns related to symptom progression and potential blindness, as well as the experience of comorbid migraine and tinnitus: “*it's a constant distraction and it makes me worry as it is progressing, day by day it gets a tiny bit worse, it makes me wonder whether I'll go completely blind one day or the after images will last for so long that I won't be able to function at all”*, and “*it has had a profound and defining impact upon my existence, and I believe I would have been quite a different person without it, as well as other health issues such as tinnitus and migraines*”.

Moreover, 8.16% of participants reflected on *medical system limitations*, citing a lack of medical understanding, diagnostic and treatment difficulties, and general discomfort with medical professionals in this space. For example, one participant stated: “*I don't even feel comfortable asking a physician or optometrist because I don't think anyone will know about it and there isn't treatment*”. A final 8.16% endorsed the final theme, that VSS is *not necessarily bad; more neutral*, which acknowledged that VSS is central to personal identity and is sometimes ignorable. Comments included, “*I'm quite neutral toward my VSS, but yeah, it's not fun having with and I miss the life and vision I had before it*”.

#### Question three—Is your Visual Snow Syndrome sometimes a good thing? What is good about your Visual Snow Syndrome?

Responses to this question generated four themes (see Tables 2.C, 5). The most common, endorsed by 54.17% of participants, was that there is *nothing good or positive* about their VSS. These responses plainly described that “*no, I hate it, there is not a good thing about it*” and that “*the only thing it taught me is that life is tough and you gotta pull through, no matter what. But a good thing? Not at all*”. Participants who were able to identify some good aspect of their VSS comprised those with both lifelong and acquired forms of the condition.

**Table 5:** Representative quotes describing what is *good* about Visual Snow Syndrome.

Theme 1. Nothing good or positive	
Nothing good or positive	° “No, I hate it, there is not a good thing about it” ° “No, never. The only thing it taught me is that life is tough and you gotta pull through, no matter what. But a good thing? Not at all” ° “No it's never good” ° “No”
Theme 2. Mental health and psychosocial wellbeing	
Attentional absorption	° “Yes, it certainly can be a good thing! I have sometimes used it as a way to distract my hyperactive mind when I have insomnia. I can't visualize in my mind's eye, so I would think that closing my eyes at night to nothing but an empty black void would be terrifying. It's somewhat comforting not being quite so close to staring into the empty unknown void of non-consciousness” ° “Sometimes the visuals are quite cool/trippy. The eye migraines can be beautiful, for instance. Random surfaces like carpets can look like they dance around” ° “Having it has helped me keep a sense of wonder and awe, that this mysterious thing that has always been with me feels somewhat magical at times, and that I adapted to it is surprising and wonderful” ° “I feel like my visual snow (and other visual effects maybe under the VS umbrella) kind of adds to the.. whimsy/fantasy flavor of my life I guess”
Resilience	° “It has made me realize how much I took for granted and it's showed me that there are way less things in my life worth being upset about” ° “As frustrating and often times debilitating as it is, I could always be afflicted with something worse” ° “Yes sometimes, I believe it strangely pushes me to work harder and keep moving despite everything going on”
Creativity	° “Yes—I see the artistic beauty in the swirls colors and patterns. I've had it all my life. I see pictures at night” ° “The feeling certain color clashes give me is great”
Community	° “It's also led me to meet people and form connections with folks I would never have had the opportunity to learn about otherwise”
Theme 3. Not necessarily good; more neutral	
Neutral response	° “There's not anything good personally however what I would say is that if my symptoms would stop progressing then I would be content as I had been for 7 years. If it would regress back to my previous baseline I couldn't complain”
Inherent to my life experience	° “It is part of who I am”
Health indicator	° “Even though it doesn't affect my life that much anymore, I obviously wish I didn't have it. Maybe the only “good” thing about it is that the severity of my symptoms tell me that I'm not living a very consistent or healthy life (I haven't slept enough, I've drank too much coffee, I have too much stress etc.)” ° “It alerts me to stressors. Physical and emotional. It intensifies when under stress. This can even cause me to remain calm during a stressful time and continue to think and process outside of the situation”
Facilitates concessions	° Nothing, otherwise, I have an excuse to not have to work full-time anymore” ° “I don't have to drive at night”
Theme 4. Functional improvements	
Vision improvements	° “I feel like it is linked to increased visual acuity. I have a high attention to detail to things like errors in page formatting etc.” ° “After images can be useful for visualizing how something should go if trying to replicate something”

*Mental health and psychosocial wellbeing* emerged once again, with 27.08% of participants describing positively valenced outcomes: that VSS provides attentional absorption by being beautiful, generating distraction and fascination, and providing a unique perspective of the world. For example, one participant respondent that “*sometimes the visuals are quite cool/trippy. The eye migraines can be beautiful, for instance. Random surfaces like carpets can look like they dance around*”. Increases in resilience, creativity and community connections were also described in responses such as, “*it has made me realize how much I took for granted and it's showed me that there are way less things in my life worth being upset about*”, and “*it's also led me to meet people and form connections with folks I would never have had the opportunity to learn about otherwise*”.

Next, 10.42% of participant responses represented the theme of that VSS was *not necessarily good; more neutral*. These responses described that VSS is manageable at the current level, and sometimes provides general health indications, for example, “*it alerts me to stressors. Physical and emotional. It intensifies when under stress. This can even cause me to remain calm during a stressful time and continue to think and process outside of the situation*”; or that VSS has become an inherent part of participant's life experience (“*it is part of who I am*”).

A final theme reflected *functional vision improvements*, including increased visual acuity and attention to detail, endorsed by a minimal number of participants (4.17%), who commented that “*I feel like it is linked to increased visual acuity. I have a high attention to detail to things like errors in page formatting etc.”* Finally, one participant responded that this question was not applicable.

## Discussion

In this first qualitative assessment of the lived experience of Visual Snow Syndrome, most participants reported that their VSS was broadly a bad thing, although the areas of life which were impacted varied between people. Importantly, though, around one third of participants were able to identify some positive aspects of their experience of VSS—and this was not limited to people with lifelong VSS. During our content analysis process, we identified remarkable agreement across questions in terms of the domains of life which are impacted by VSS: the same themes repeated across all three questions considered here. Rather than seeking to explain why individuals differ in their experience of VSS, our aim was to characterize the range of experiences reported by people living with the condition. To this end, this discussion will address key themes from our results, rather than moving through each question sequentially.

These findings demonstrate the unique contribution of qualitative approaches to understanding VSS. While quantitative studies have established associations between VSS and psychiatric symptoms, quality of life, and symptom severity, they cannot readily capture how individuals interpret, adapt to, and make meaning of living with the condition. By documenting the breadth and variability of patient experiences, the present study complements existing quantitative literature and identifies patient priorities and support needs that may inform both future research and clinical care.

### Mental health and psychosocial wellbeing

Impacts to mental health and psychosocial wellbeing were the core component of our participants' lived experience of VSS. These impacts were generally negative, and manifested across several domains including mood and emotions, cognition, sleep, daily life functioning, and inter-personal relationships. Of these, mood and emotional outcomes (i.e., anxiety, fear, sadness, hopelessness, and fatigue) were the most commonly reported “bad” aspects of living with VSS, whether experienced persistently or intermittently. Multiple participants noted that their mental health shared a bi-directional and even cyclical link with visual symptoms, such that the emergence and presence of either set of symptoms exacerbates the other. These findings offer the first lived experience testimony to support quantitative evidence that psychiatric symptoms, particularly higher rates of anxiety and depression, are prevalent in VSS (3, 21).

In addition to affective symptoms, several participants described dissociative experiences of “*feeling like I'm in a dream or I'm not real*” and that “*everything feels fake*”, or even directly stated that their VSS causes depersonalization and derealization. Solly et al. (3) also reported the presence of these symptoms in people with VSS, but cautioned that the understanding of these lesser-known phenomena remains limited. As evidenced in our participants' responses, people may not know that their experience is a defined psychological symptom, or may self-attribute this terminology without appropriate clinical guidance. In order to fully support people with VSS to understand and manage the complexities of their condition, it is essential that health professionals assess for mental health symptoms – and educate their patients on them.

Participants also described numerous psychosocial impacts of their VSS. Sleep was a commonly reported issue, with participants describing difficulties falling asleep and staying asleep. One participant specified that falling asleep was challenging due specifically because their visual phenomena were more pronounced at night. Other participants noted that poor sleep worsens their visual phenomena. This is particularly interesting in light of recently published results indicating significantly worse subjective sleep quality (scores on the Pittsburgh Sleep Quality Index) among people with diagnosed VSS compared to healthy controls (22). Furthermore, cognitive difficulties such as overstimulation, distraction, and brain fog affect most daily activities for a majority of participants. Similar findings of subjective cognitive impairments have been reported in a number of quantitative studies, but there has so far been limited research investigating cognition objectively (23, 24). One psychophysical study has shown no significant difference between people with VSS and controls on common tests of directed attention (25).

Regardless of whether the cognitive impairments reported by our participants are perceived or measurable, they have a substantial impact on quality of life. As a result of challenges related to sleep and cognition, many participants reported substantial changes and accommodations, often experiencing significant losses in daily life. These outcomes are major: career options are limited, learning is taxing, and typical behaviors such as driving, using digital devices, and reading are challenging, if not impossible. A distinct sense of isolation and shame also emerged in these descriptions, indicating the complex, far-reaching and often invisible burdens of VSS. This is an important consideration for clinicians: when determining options for managing participants' VSS, the impacts on their daily lives should not be overlooked. Regardless of whether can be provided with sleep hygiene or cognitive difficulties, it is important to acknowledge the broader implications for participants' wellbeing, and not to focus only on alleviating visual symptoms.

A small proportion of participants endorsed positive psychosocial outcomes as “good” aspects of VSS. They acknowledged that VSS can provide pleasing distraction and generate fascination, increased creativity, and a unique perspective of the world. Costa et al. (15) recently defined the potential for visual snow to create “attentional absorption”, hypothesizing that a hyperexcitability of the visual cortex may render those with visual snow more engrossed in perceptual and imaginary experiences. Our results support this theory, and show that some people with VSS find their visual phenomena to impact their lives in enjoyable ways, although this was not the majority experience. Some participants also noted that living with VSS increases their resilience. This adaptive psychological framework and protective factor could be capitalized on in clinical settings, as indicated by the promising findings of a recent pilot trial of mindfulness-based cognitive therapy for VSS (26).

It is notable that these mental health and psychosocial presentations remained impactful despite participants' normal DASS-21 scores at the time of participation. We speculate that the descriptions given in this study reflect the dynamic nature of mental health and chronic illnesses over time, as well some discordance between the ongoing personal impacts of mental health difficulties and what is deemed clinically significant. It is possible that people with VSS experience some degree of chronic sub-threshold psychological distress that is normalized and internalized as a component of their VSS illness course. Similar effects have been shown in migraine, where a high proportion of patients report excessive anxiety, worry and sadness, and a mindset toward these symptoms, without indicating a clear psychiatric comorbidity (27, 28). Ultimately, our findings emphasize the need for more integrative treatments for VSS, that not only seek to ameliorate visual symptomatology but which also minimize psychological distress, leverage resilience, and promote wellbeing (24).

### Functional vision impacts

Our participants reported a range of impacts related to functional vision, including both perceived improvements and perceived reductions in visual quality. For example, while some participants reported that they felt their visual clarity and depth perception had worsened since the onset of their VSS, others felt that VSS improved their attention to detail. Importantly, the level of detail most participants provided did not make it possible to determine whether any problematic changes to functional vision that they described might be better attributed to alterations in cognitive function, such as brain fog. This may be an interesting direction for future research.

The most commonly reported impact on functional vision related to visual comfort in different light levels. Participant responses revealed substantial inter-individual differences in the optimal light levels. Some participants struggled with bright light, whereas others struggled with low light levels, and sometimes participants reported that both bright and low light levels caused discomfort or difficulty seeing. Complaints related to artificial light (i.e., hospital lighting, office lighting) causing discomfort were common, as were problems appreciating nature or the outside world due to discomfort caused by sunlight. These findings align with the findings of Puledda et al. (29), who found a significant effect of light levels on reported density of visual snow, with both nighttime and fluorescent lights being identified as problematic. Additionally, 40 participants reported photophobia on the MVSQ and six described this a “bad” aspect of their VSS. We have previously shown photophobia is associated with the perceived severity of VSS symptoms (12), and Eren et al. (30) showed that it exists in people with VSS at levels comparable to those of migraine patients during an attack.

Together, these results suggest that much of the quantitative literature accurately captures aspects which impact functional vision in people with VSS. However, one factor which comes through more clearly in this study compared to the rest of the literature is the sense of loss which sometimes accompanies these changes. For example, participants' descriptions of challenges with sunlight were often characterized by the sadness and deprivation, as many found that they were no longer able to enjoy nature or the outside world anymore. This is a notable loss for quality of life, as well as a problem for functional vision.

These findings have some clear implications for clinical practice. Given the wide-ranging functional vision impacts participants describe, especially regarding which light levels are comfortable, it is important for clinicians to note that tinted lenses may not result in improved quality of life. While there is preliminary evidence indicating that such lenses may be effective for some patients (31, 32), our findings clarify that triggers for visual discomfort vary substantially between people—and even sometimes within people, across time. Treatment options related to functional vision should therefore be tailored to the individual.

### The dynamic course of VSS

So far, there has been limited research into the longterm impacts of VSS, including the extent to which symptoms or their impacts can change over time, although recent papers indicate that VSS symptoms may fluctuate over periods from weeks to years (9, 14, 29). Although we did not specifically probe longitudinal changes, our participants' responses indicate that VSS (including visual snow, other key diagnostic symptoms, and issues related to mental health and psychosocial wellbeing) are highly changeable over time. Many participants commented that their VSS has become normal for them, or that it has become a mild annoyance (implying that it was once not normal or had more negative impacts). Responses to our question about whether VSS is ever a good thing indicated that six participants viewed their VSS as broadly neutral, although most of these participants also indicated that they did not always view their VSS this way. This suggests that it is possible to adapt to VSS over time, a fact which may be reassuring in clinical settings.

In response to our question about why VSS is a bad thing, some participants identified specific factors which would cause their VSS experiences to worsen, including sleep deprivation, stress, and the onset of migraine attacks. Migraine is highly prevalent in VSS and may influence aspects of lived experience, including photophobia, emotional wellbeing, and symptom fluctuation. Because the present study was not designed to compare participants with and without migraine, we cannot determine the extent to which individual themes are specifically attributable to VSS vs. comorbid migraine. Future qualitative work should directly compare these groups.

Many participants described instances of increased symptom severity or impact, often in relation to other key themes such as functional vision (e.g., lighting) and psychosocial wellbeing. For example, in response to our question about the impact of visual snow, one participant stated that their visual snow had only become “problematic” in the last few months, because they took a new job in an office with suboptimal lighting conditions. Three participants stated that one of the “good” things about VSS is that it provides them with an indication of their general health and wellbeing: one participant said, “*the severity of my symptoms tell me that I'm not living a very consistent or healthy life*”, where another reported, “*it alerts me to stressors. Physical and emotional. It intensifies when under stress. This can even cause me to remain calm during a stressful time*”. Several participants also indicated that one of their greatest fears is symptom progression, whether or not this has eventuated for them.

Together, these findings paint a picture of a condition that can change substantially from day to day, and which is heavily impacted by both external factors such as light level, and internal factors including increased stress levels, other instances of poor mental health, and migraine attacks. Broadly, our results align with the few longitudinal studies on VSS, showing that people's symptoms are always present to some degree, but do change in presentation, frequency, and impact across time (9, 14, 29). While it is clear that more work is needed to track the time course of changes to VSS symptoms and to understand whether symptom progression can be prevented, it is also important for clinicians to note that there are numerous existing and well-documented interventions which could help address some of the common triggers for VSS symptoms worsening.

In clinical practice, the most impactful way of enhancing the wellbeing of people with challenging cases of VSS may be to focus on improving general mental and physical health and wellbeing. The data presented here indicate that this may be the most effective means of alleviating the burden of VSS for those who are suffering, particularly while treatments addressing functional vision impacts are still in their infancy (32, 33).

### Medical system limitations

A minority of participants indicated that the worst things about their experience of VSS related to the medical system directly. Notably, participants described distress at a lack of viable treatment options, as well as poor experiences with medical professionals in the past. One participant specifically described feeling psychologically unsafe describing their symptoms to doctors. While there has been no systematic research (either qualitative or quantitative) on VSS patients' interactions with the medical system to date, several papers highlight how VSS is challenging to diagnose and that patients are often dismissed as malingering (3, 5, 34, 35). Our findings align with this, and this may go some way toward explaining the extremely high rate of self-diagnoses in our study, as well as the growing number of online support groups and communities for people with VSS. Our above-described psychosocial wellbeing findings indicate that many people with VSS feel isolated or ashamed of their condition, and it is not hard to imagine that this could be exaggerated when clinicians are dismissive of lived experience. It is important for both researchers and clinicians to bear in mind the stigma associated with VSS when working with people with the condition. Our results make it clear that the words doctors use to describe VSS can impact how patients perceive their experience, whether the doctors are aware of the condition or not.

### Participant-related considerations

In assessing the findings of this study, it is important to consider various aspects of how our sample was derived: our participants were recruited via online support groups and discussion forums for people with VSS. This potentially introduces two participant-related limitations to our work. First, there is a small possibility that some participants may not have clinically-defined VSS; and second, it is possible that some degree of self-selection bias has resulted in people with more severe presentations taking part in our study, precluding more neutral experiences.

Regarding the first issue, our participants all self-identified as experiencing VSS, and many reported formal medical diagnoses. As none of the researchers involved in this study are clinicians, we were unable to independently verify participants' reported diagnoses. However, we carefully matched participants' reported symptoms and symptom frequency against the established ICHD diagnostic criteria for VSS, and 38 of 49 participants seemingly met these criteria. Of the remainder, one participant reported a formal VSS diagnosis although their visual snow was intermittent; five experienced visual snow in the absence of sufficient additional symptoms to be categorized formally as experiencing VSS; and in five cases, participants reported the use of recreational drugs before the onset of their symptoms, potentially indicating HPPD. Our approach to categorization was cautious, but we included all participants' respsonses for analysis regardless of category. Notwithstanding the possibility of a more complex explanation for their symptoms, all our participants experienced many perceptual phenomena associated with VSS—and importantly, they all *believed* they had VSS. Given this study presents findings related to lived experience, the fact that our participants all self-identified as having VSS means that their experiences should be informative to future researchers and clinicians regardless of whether a fuller diagnostic process would result in a VSS diagnosis.

Regarding the second issue, people whose experience of VSS has never required them to seek either medical or psychosocial support are unlikely to participate in the kinds of online support groups from which we recruited. This may go some way to explaining why the vast majority of our participants reported that the impacts of their VSS were broadly bad. We attempted to mitigate for this to some degree by specifically targeting groups for people who had lifelong VSS in addition to support groups populated mostly by those whose VSS had recently onset. However, this remains a limitation of our work.

### Study limitations

Our findings should be interpreted in line with the limitations of this study. First, our qualitative questions were asked within a survey, alongside quantitative scales, and phrased with some degree of context that could introduce bias to participant responses. However, our question about the *impacts* of VSS was open-ended and we were sure to balance more direct probing of *bad* aspects with equal opportunity to describe the *good*. Given no other qualitative lived experience perspectives of VSS currently exist, this study provides essential context to the quantitative works that continue to emerge.

Second, whilst participant responses to each question were systematically categorized into distinct themes, there was remarkable consistency in the domains of health, vision and functioning endorsed, and in many cases participant responses referenced multiple themes. It is likely that these themes share some interdependence, and future research could seek to characterize directionality and/or causality of their relationship to VSS. We also chose not to compare the experiences of people with and without migraine, or with and without mental health or psychiatric comorbidities, separately. This may have influenced the themes which emerged in our findings. Our findings reveal that both migraine and mental health considerations, as well as a range of other health concerns, are inherent to people's lived experience of VSS, and separating each of these concerns out would have both reduced sample size considerably and potentially indicated differing subtypes of VSS, which is beyond the scope of this study.

Finally, we note that we could not query more specific participant demographic variables such as location, ethnicity, occupation, etc. due to ethics requirements. These details may be useful to further clarify the factors that contribute to the lived experience of VSS.

### Conclusion

This study presents a comprehensive exploration of the lived experience and subjective impacts of VSS. It highlights first-hand evidence that VSS is not a universally negative experience, but can be one that can bring meaning, fascination, and a unique perspective to those who live with it. However, for a majority of our sample, VSS introduces substantial mental health and psychosocial wellbeing challenges, as well as functional vision impairments to their day-to-day life. Our results also emphasize that the lived experience of VSS varies hugely, both from person to person, and within the same person, but across time. To this end, clinicians and researchers should be careful to consider individualized approaches going forward. A more holistic understanding and treatment of these multi-dimensional outcomes is therefore a distinct and urgent priority for the long-term support of people living with VSS.

## Data Availability

The raw data supporting the conclusions of this article will be made available by the authors, without undue reservation.
